# A Room-Temperature, High-ppb-Level NO Gas Sensor Based on Pt/WO_3_ Co-Decorated Carbon Nanofibers Towards Asthma-Relevant Breath Analysis Application

**DOI:** 10.3390/s26031069

**Published:** 2026-02-06

**Authors:** Shanshan Yu, Xingyu Liu, Jinshun Wang, Qiuxia Li, Yuhao Pang, Lixin Zhang, Chen Yang, Qingkuan Meng, Cao Wang, Qiang Jing, Jingwei Chen, Bo Liu

**Affiliations:** 1 Laboratory of Functional Molecules and Materials, School of Physics and Optoelectronic Engineering, Shandong University of Technology, 266 Xincun Xi Road, Zibo 255000, China; 13780908162@163.com (S.Y.); xingyuliu202211@163.com (X.L.); 19546304631@163.com (J.W.); 19862540463@163.com (Q.L.); ppyh123gg@163.com (Y.P.); zzhanglixin7@163.com (L.Z.); yc2092694103@163.com (C.Y.); qkmeng@sdut.edu.cn (Q.M.); wangcao@sdut.edu.cn (C.W.); 2 School of Mathematics and Physics, Xi’an Jiaotong-Liverpool University, Suzhou 215123, China; bo.liu@xjtlu.edu.cn

**Keywords:** NO gas sensor, room-temperature sensing, Pt/WO3-decorated carbon nanofibers, electrospinning

## Abstract

A chemiresistive nitric oxide (NO) gas sensor based on Pt/WO_3_ co-decorated carbon nanofibers (CNFs) was fabricated using a simple and scalable electrospinning process. This sensor demonstrates high-ppb-level NO detection at room temperature (25 °C), with an experimentally demonstrated detection limit of 100 ppb. It exhibits rapid response, good signal repeatability, excellent batch-to-batch reproducibility, and high selectivity toward NO. Compared with previously reported NO sensors, this work highlights the integration of Pt and WO_3_ within a conductive CNF network, enabling room-temperature NO detection down to 100 ppb using a simple chemiresistive architecture. In addition, preliminary sensing tests were conducted using dried simulated breath samples prepared by introducing exogenous NO into exhaled breath from healthy volunteers, demonstrating the sensor’s capability to resolve different NO levels in a complex breath-related background. Owing to its reliable performance and cost-effective fabrication, the sensor holds potential as a NO sensing platform, providing a materials-level basis for future breath NO analysis and other related applications.

## 1. Introduction

Research in metabolomics has shown that small molecules (<1500 Da) are present in breath, urine, saliva, tissue, blood, other body fluids, and cells [1]. Among these matrices, exhaled breath is particularly notable because it contains volatile organic compounds (VOCs) that can serve as biomarkers for disease detection, enabling noninvasive diagnosis and supporting personalized medicine [1]. In some settings, breath analysis can even identify disease at earlier stages than conventional diagnostic methods [2]. To date, 874 VOCs have been identified in human exhaled breath [3]. Asthma is a heterogeneous chronic respiratory disease affecting all ages and ethnicities and imposes a substantial global burden—approximately 358 million people worldwide [4,5]. Type 2 inflammation is common in asthma and is present in up to 80% of corticosteroid-naïve patients [6]. Airway inflammation activates IL-4/IL-13→iNOS pathways, increasing nitric oxide (NO) production by airway epithelial cells [7,8]. NO diffuses into exhaled breath, and the fractional exhaled NO (FeNO) serves as a surrogate biomarker of Type 2 inflammation [8]. Consequently, FeNO measurement has become a valuable tool for diagnosis and monitoring of asthma.

Properly engineered carbon-based materials, when combined with functional metal oxides or catalytic components, can provide favorable charge transport pathways and accessible active sites for NO sensing. Their high electrical conductivity supports efficient charge transport [9,10], while surface functionalization can promote NO adsorption and improve sensitivity [9,11]. Among these, carbon nanofibers (CNFs) are particularly attractive because they combine high conductivity, mechanical robustness, thermal stability, and large specific surface area with facile processing and controllable synthesis/functionalization [12]. Tungsten trioxide (WO_3_), an n-type semiconductor, is widely employed for NO sensing [13,14,15,16,17,18,19]. For example, Cai et al. grew single-crystalline WO_3_ nanowires on FTO and achieved high NO sensitivity and selectivity [20]; Moon et al. synthesized one-dimensional villi-like WO_3_ on SiO_2_/Si, which exhibited strong NO responses at 200 °C [21]. Noble-metal doping can further improve the performance of the sensor by introducing surface defects, increasing active-site density, and providing catalytic activity [22,23,24,25,26]. Highly sensitive sensors have been reported using Au [26], Ag [5,27], Pt [28,29], Pd [30,31,32], and Rh [32,33,34]; among these, Pt is especially effective owing to strong electronic sensitization that enhances sensitivity and reduces operating temperature [35,36,37]. Electrospinning is a simple, effective, and low-cost method for synthesizing nanofibers then fabricating gas sensors [38,39,40,41]. In this study, we fabricated a NO sensor based on electrospun Pt/WO_3_ co-decorated CNFs. The sensor achieved an experimentally determined detection limit of 100 ppb at room temperature (25 °C) and exhibited a rapid response, good repeatability, and high selectivity toward NO. Under controlled laboratory conditions, these results demonstrate the potential of the proposed sensor as a room-temperature NO sensing platform, providing a materials-level basis for future studies related to asthma-relevant breath NO analysis.

## 2. Experimental Section

(1) Preparation of Pt/WO_3_-CNF via Electrospinning

**Figure 1:**
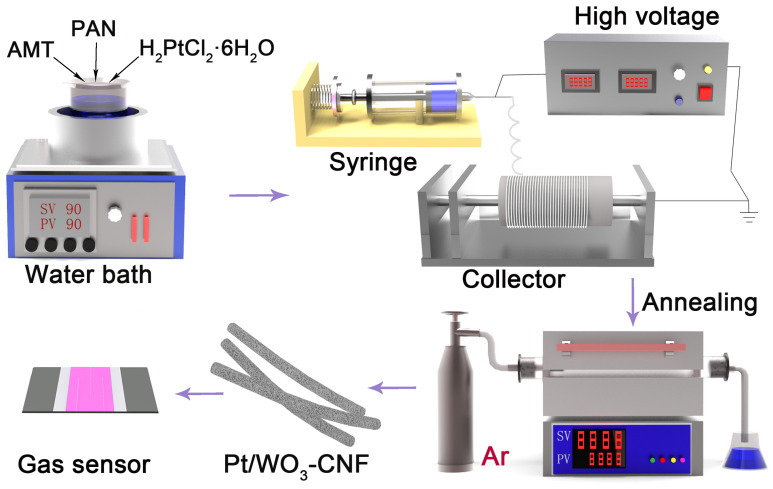
Schematic diagram of the fabrication process for the sensing material and the gas sensor device.

The synthesis process is illustrated in Figure 1. Typically, 1 g of polyacrylonitrile (PAN) was dissolved in 10 mL of N,N-dimethylformamide (DMF) and magnetically stirred in a water bath at 90 °C for 1 h until a transparent, homogeneous, light-yellow polymer solution was obtained. Subsequently, 20 mg of hexachloroplatinic acid (H_2_PtCl_6_·6H_2_O) and a predetermined mass of ammonium metatungstate (AMT) were slowly introduced into the solution. The mixture was then stirred at 90 °C for 2 h to ensure complete dispersion and dissolution, yielding a stable precursor solution.

The precursor was loaded into a syringe and electrospun under the following conditions: an applied voltage of 25 kV and a feed rate of 0.015 mL min^−1^. The collected nanofiber mat was dried in air at 60 °C for 4 h to remove residual solvent. Subsequently, the mat was transferred to a tubular quartz furnace and annealed in air under controlled heating conditions. The temperature was first increased to 220 °C at a rate of 1 °C min^−1^ and maintained for 1 h for pre-oxidation. It was then raised to 480 °C at 5 °C min^−1^ and held for 1 h to enable AMT decomposition and partial WO_3_ formation. Finally, the atmosphere was switched to argon, and the temperature was elevated to 650 °C at 2 °C min^−1^ and maintained for 1 h to achieve carbonization. After cooling naturally to room temperature, the Pt/WO_3_–CNF composite was obtained.

(2) Fabrication of the Sensor Based on Pt/WO_3_-CNF

The synthesized Pt/WO_3_–CNF powder was transferred to an agate mortar. An appropriate amount of anhydrous ethanol was added, and the mixture was thoroughly ground to obtain a homogeneous paste. This paste was uniformly coated onto a commercial Al_2_O_3_ substrate with Ag–Pd interdigitated electrodes to fabricate the sensing device. The device was then placed in an oven at 90 °C for 6 h to remove residual solvent and improve film adhesion and structural stability.

(3) Gas sensing measurement

Gas sensing measurement was evaluated using a CGS-4TPS four-channel static testing system (custom-built by Beijing Elite Tech Co., Ltd., Beijing, China). This system is composed of temperature control system (from room temperature to 500 °C, accuracy ±1 °C), a gas mixing and distribution module, a probe positioning system, a data acquisition system, and software for resistance measurement. The testing apparatus and procedures were identical to those used in our previous study [34,42]. The target gas was injected into the sealed test chamber using a micro-syringe.

Gas sensing measurements were performed using a CGS-4TPS four-channel static testing system (custom-built by Beijing Elite Tech Co., Ltd.). The system comprises a temperature-control unit (room temperature to 500 °C; accuracy ±1 °C), a gas-mixing and distribution module, a probe-positioning stage, a data-acquisition unit, and resistance-measurement software. The apparatus and procedures were identical to those in our previous study [34,42]. The target gas was introduced into the sealed test chamber using a microsyringe. For gases supplied directly in the gas phase, the injected volume was calculated as $V_{\mathrm{target}\;\mathrm{gas}}=C\times V_{s}$, where *C* is the desired gas concentration and $V_{s}$ is the chamber volume. For the target gases that are liquid at room temperature, the concentration C achieved in the chamber was calculated using

(1) $$C=\frac{\rho_{\mathrm{mixed}\;\mathrm{liquid}}\times V_{\mathrm{mixed}\;\mathrm{liquid}}\times d/M_{\mathrm{target}\;\mathrm{liquid}}}{P_{0}V_{\mathrm{chamber}}/\left(RT_{0}\right)}$$

where $\rho_{\mathrm{mixed}\;\mathrm{liquid}}$, $V_{\mathrm{mixed}\;\mathrm{liquid}}$ are the density and volume of the prepared liquid mixture (solvent + target liquid), *d* is the mass purity of the target liquid, $M_{\mathrm{target}}$ is its molar mass, $P_{0}$ is the standard atmospheric pressure, $V_{\mathrm{chamber}}$ is the test-chamber volume, *R* is the ideal-gas constant, and $T_{0}$ is the ambient temperature.

During testing, the system automatically recorded the sensor resistance (before and after gas injection), the operating temperature, and the ambient humidity. The sensor response *V* was defined as the ratio of the resistance in air, $R_{a}$, to that in the target-gas atmosphere, $R_{g}$, i.e., $V=R_{a}/R_{g}$ Experimental results indicated that the Pt/WO3–CNF sensor, doped at $W_{{\mathrm{WO}}_{3}}$/$C_{\mathrm{CNF}}$ = 1.07 at%, and Pt/$C_{\mathrm{CNF}}$ = 0.07 at% (atomic ratio), exhibited the best gas-sensing performance; subsequent tests were therefore conducted primarily with this composition.

## 3. Results and Discussion

### 3.1. Characteristics of Sensing Materials

**Figure 2:**
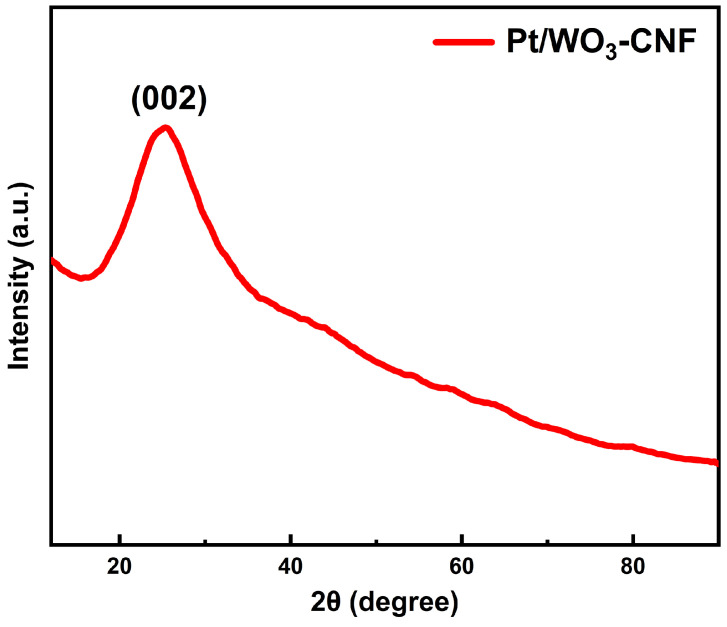
XRD patterns of the sensing material, based on which the sensor presents the best gas sensing performance.

Figure 2 presents the XRD patterns of the optimal Pt/WO_3_–CNF sensing material. A dominant diffraction peak for the carbon nanofibers is observed at approximately 24.8°, corresponding to the (002) plane of graphitic carbon [43]. This distinct peak indicates relatively high crystallinity, implying a well-ordered atomic structure that is generally advantageous for gas-sensing applications. In contrast, no characteristic peaks associated with Pt or WO_3_ are observed, which is attributable to their low contents in the composite. In addition, the elemental composition and chemical states of the Pt/WO_3_–CNF composite were further analyzed by XPS, as shown in Figure S1.

**Figure 3:**
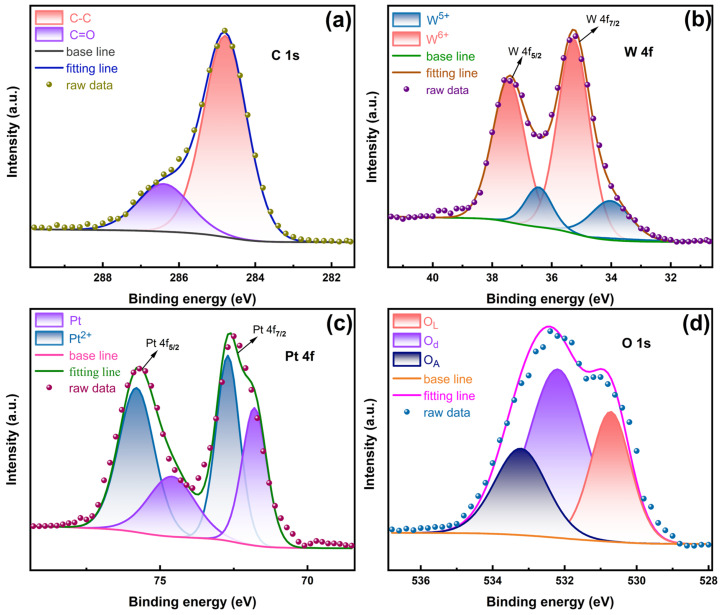
(**a**–**d**) The core-level XPS spectra of C 1s, W 4f, Pt 4f, and O 1s for the Pt/WO_3_–CNF sensing material.

To further examine the chemical composition and valence states of the composite, X-ray photoelectron spectroscopy (XPS) was conducted. Figure 3a presents the C 1s core-level XPS spectrum, where the peaks at 284.8 eV and 286.5 eV are assigned to C–C and C=O bonds, respectively [43]. Figure 3b shows the W 4f spectrum, which consists of two sets of doublets. The peaks at 35.3 eV and 37.2 eV correspond to W 4f_7/2_ and W 4f_5/2_ of W^6+^, whereas the peaks at 34.2 eV and 36.6 eV are attributed to W^5+^ [44,45,46]. The detection of W^5+^ species, which are commonly associated with oxygen vacancies, indicates the presence of defect states that may enhance gas-sensing performance, particularly for NO and NO_2_ detection [47,48]. While only a single WO_3_ loading was investigated in this study, these oxygen vacancies are likely one of the factors contributing to the observed NO sensing performance [49]. As illustrated in Figure 3c, the Pt 4f spectrum reveals two distinct oxidation states of Pt. The Pt 4f_7/2_ peaks at 71.8 eV and 72.7 eV are assigned to Pt^0^ and Pt^2+^, respectively, confirming the coexistence of metallic Pt and PtO [50]. Metallic Pt (Pt^0^) facilitates charge transport and provides catalytic active sites [51], whereas Pt^2+^ is generally associated with enhanced NO adsorption and oxidation. The simultaneous presence of both species suggests a synergistic effect that contributes to improved NO-sensing performance [52,53]. Figure 3d shows the O 1s spectrum, which can be deconvoluted into three components. Peaks at 530.7 eV, 532.2 eV, and 533.2 eV are assigned to lattice oxygen (O_L_), defect-related oxygen (O_d_), and adsorbed oxygen species(O_*A*_), respectively [54,55]. The presence of adsorbed oxygen species and oxygen vacancies are widely recognized to enhance the gas sensing performance of the sensor [56,57].

**Figure 4:**
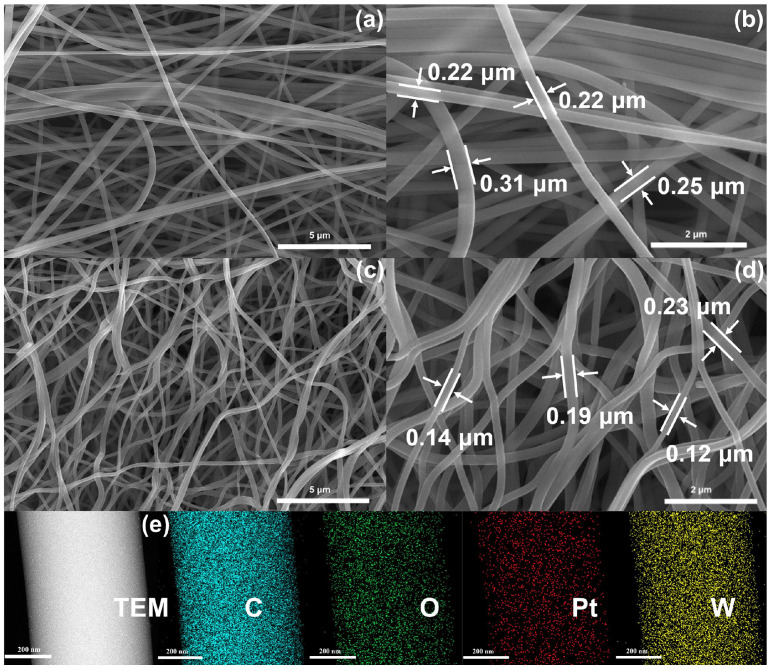
(**a**,**b**) Low- and high-magnification SEM images of the Pt/WO_3_-CNF composite before calcination. (**c**,**d**) Low- and high-magnification SEM images of the Pt/WO_3_-CNF composite after calcination. (**e**) EDS mapping of the Pt/WO_3_-CNF composite.

**Figure 5:**
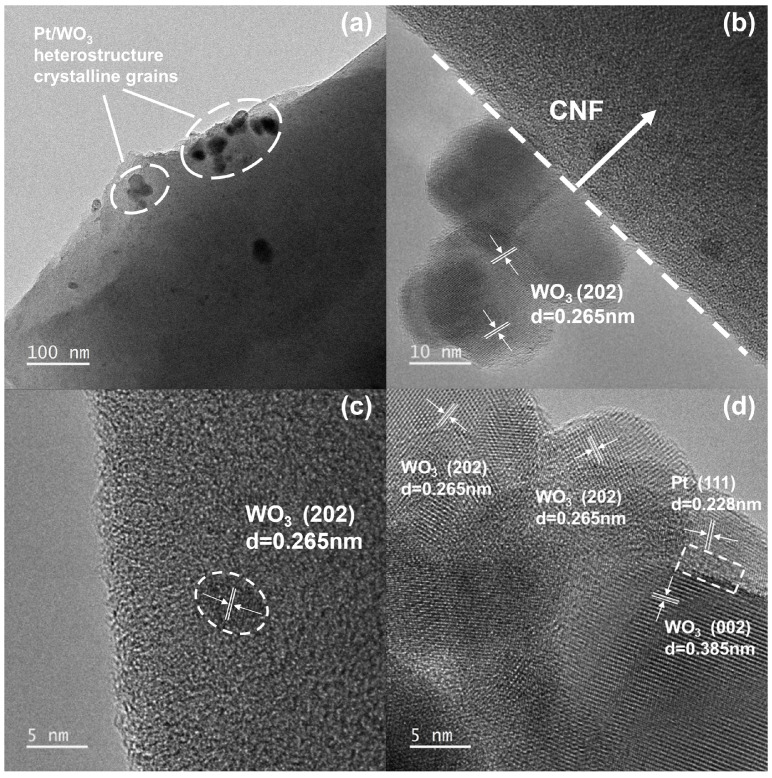
TEM images of the Pt/WO_3_-CNF composite, where nanoparticles of either Pt or WO_3_ (**a**), and CNF (**b**) are highlighted. (**c**) HRTEM image of a single carbon nanofiber revealing crystallized carbon domains, with a WO_3_ crystalline nanoparticle also visible. (**d**) HRTEM image of the Pt/WO_3_ heterostructure decorated on the surface of a single carbon nanofiber, where a WO_3_–Pt heterostructure is outlined by a dashed rectangle.

Figure 4a,b show low- and high-magnification SEM images of the Pt/WO_3_–CNF composite before calcination. The nanofibers display smooth surfaces with diameters ranging from 0.22 to 0.31 μm. Figure 4c,d present the corresponding images after calcination, where individual fibers become thinner, with diameters of 0.12–0.23 μm. Figure 4e shows EDS elemental maps of the Pt/WO_3_–CNF sensing material, indicating that C, O, Pt, and W are homogeneously distributed throughout the fibers; this uniformity suggests that Pt and the WO_3_ phase are likewise uniformly dispersed. Figure 5a,b show TEM images of the Pt/WO_3_-CNF composite, where nanoparticles of either Pt or WO_3_ in Figure 5a, and CNF in Figure 5b are highlighted. As shown in Figure 5b, three WO_3_ nanoparticles are observed on the CNF surface. Figure 5c presents an HRTEM image of a single carbon nanofiber bearing a crystalline WO_3_ nanoparticle, where the presence of crystallized carbon domains are also observed. Figure 5d shows an HRTEM image of a nanoparticle cluster on the CNF surface, in which individual particles are assigned to WO_3_ and Pt; a WO_3_–Pt heterostructure is visible and outlined by a dashed rectangle. The lattice fringes of WO_3_ and Pt are consistent with the corresponding PDF cards (Nos. 01-083-0950 and 00-004-0802), confirming the phase identification. To further verify the crystallinity and phase structure of the Pt/WO_3_–CNF composite, the selected-area electron diffraction (SAED) pattern is provided in Figure S10.

### 3.2. Gas Sensing Performance

**Figure 6:**
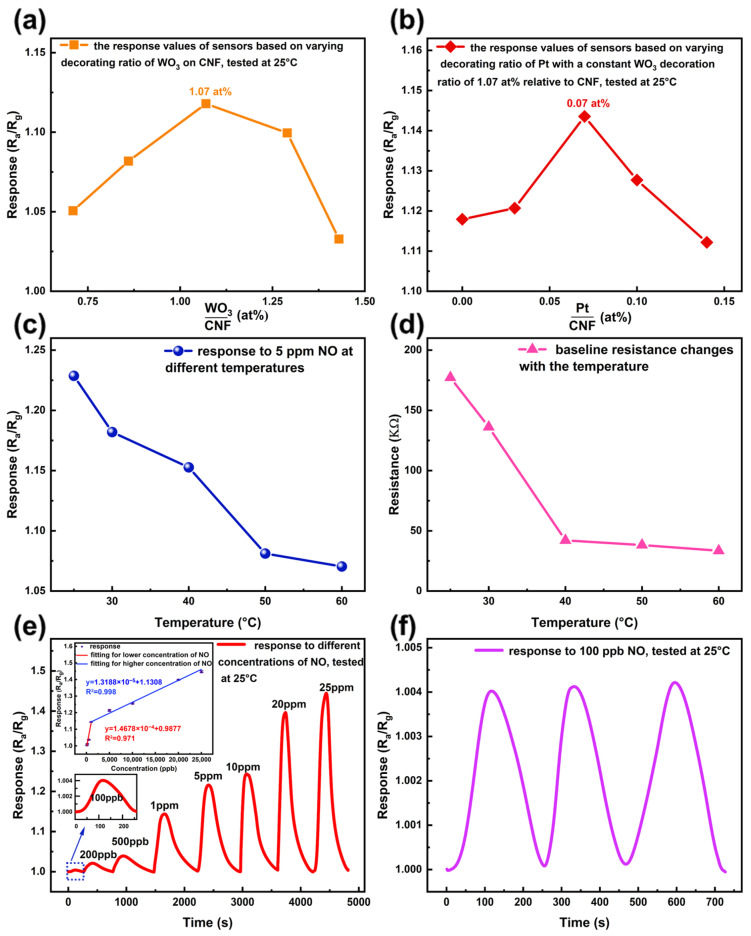
(**a**) WO_3_ decorating ratio-dependent response values of the sensor based on WO_3_-decorated CNF. (**b**) Pt decorating ratio-dependent response values of the sensor based on Pt and WO_3_ co-decorated CNF, with a constant WO_3_ decoration ratio of 1.07 at% relative to CNF. Temperature-dependent response values (**c**) and temperature-dependent baseline resistance (**d**) of the sensor. (**e**) Sensor responses to varying NO concentrations, measured at room temperature (25 °C). The inset presents the linear fitting of the concentration-dependent response values in (**e**). (**f**) Repeatability test of the sensor at the detection-limit concentration of NO (100 ppb).

To obtain a sensor with the best gas-sensing performance, the decoration ratios of WO_3_ and Pt were systematically optimized. Figure 6a shows the responses of WO_3_–decorated CNFs as a function of the WO_3_ decoration ratio. The sensor with a WO_3_ decorating of 1.07 at% exhibited the highest response, indicating that the optimal WO_3_/CNF ratio is 1.07 at%. Building on this result, the optimal Pt decoration ratio was investigated while maintaining a constant WO_3_ decoration of 1.07 at%. Figure 6b displays the Pt–decoration–dependent responses of the sensor based on Pt/WO_3_ co-decorated CNFs, with a fixed WO_3_ decoration ratio of 1.07 at% relative to CNF. The best performance toward NO was obtained with co-decoration of 1.07 at% WO_3_ and 0.07 at% Pt (relative to CNF). All subsequent measurements were performed using this optimized sensor. These findings suggest that appropriate Pt and WO_3_ decoration significantly enhance sensing performance, whereas excessive decoration leads to nanoparticle agglomeration. Such agglomeration reduces the number of accessible active sites on the Pt and WO_3_ surfaces, impeding interactions between NO molecules and adsorbed oxygen species and thereby lowering the sensor’s sensitivity and overall performance [58].

The temperature-dependent response of the optimized sensor to 5 ppm NO is shown in Figure 6c. The response decreases monotonically with increasing temperature, with the maximum sensitivity observed at room temperature (25 °C). The sensing mechanism generally involves two steps: (i) adsorption of gas molecules onto the sensing surface and (ii) redox reactions between the gas and chemisorbed oxygen species [59]. Both processes are strongly temperature dependent. At low temperatures (25 °C), chemisorbed oxygen species dominate the surface chemistry by capturing conduction-band electrons, thereby increasing the probability of adsorption and reaction of target molecules such as NO. As the temperature rises, however, the desorption rate of oxygen species accelerates, diminishing the number of active sites. Furthermore, higher temperatures also promote NO desorption, further reducing the response [60,61]. At 25 °C, a balance between adsorption and desorption is achieved, resulting in optimal sensing performance [62]. Beyond this temperature, the desorption of both NO and O_2_ exceeds their adsorption, collectively impairing the sensor response. Figure 6d shows the temperature-dependent baseline resistance of the sensor, which decreases with increasing temperature. This behavior can be ascribed to the p-type nature of CNF (holes as majority carriers) [9]. With increasing temperature, more electrons are thermally excited to higher energy states, leaving behind additional holes in the valence band. Consequently, the hole concentration rises, further lowering the resistance. The responses of the sensor to NO concentrations ranging from 100 ppb to 25 ppm at 25 °C are presented in Figure 6e. The experimentally demonstrated detection limit of the sensor is 100 ppb. The inset displays the corresponding concentration–dependent response values with error bars, revealing two distinct linear regimes: 100 ppb–1 ppm and 1–25 ppm, with an inflection point at 1 ppm. In addition, the limit of detection (LOD) was statistically estimated using the widely adopted 3σ/slope criterion, yielding a value of approximately 30 ppb, which is lower than the experimentally demonstrated detection limit. This difference between the theoretical and experimental LOD is commonly reported in chemiresistive gas sensors, as the theoretical LOD is derived from idealized signal statistics and does not fully account for practical factors such as background noise, signal fluctuations, and experimental conditions. Finally, Figure 6f demonstrates the repeatability test of the sensor toward the detection-limit concentration (100 ppb) of NO. The good signal reproducibility of the sensor even at the lowest measurable level is confirmed. The response parameters of the sensor is summarized in Table 1.

**Figure 7:**
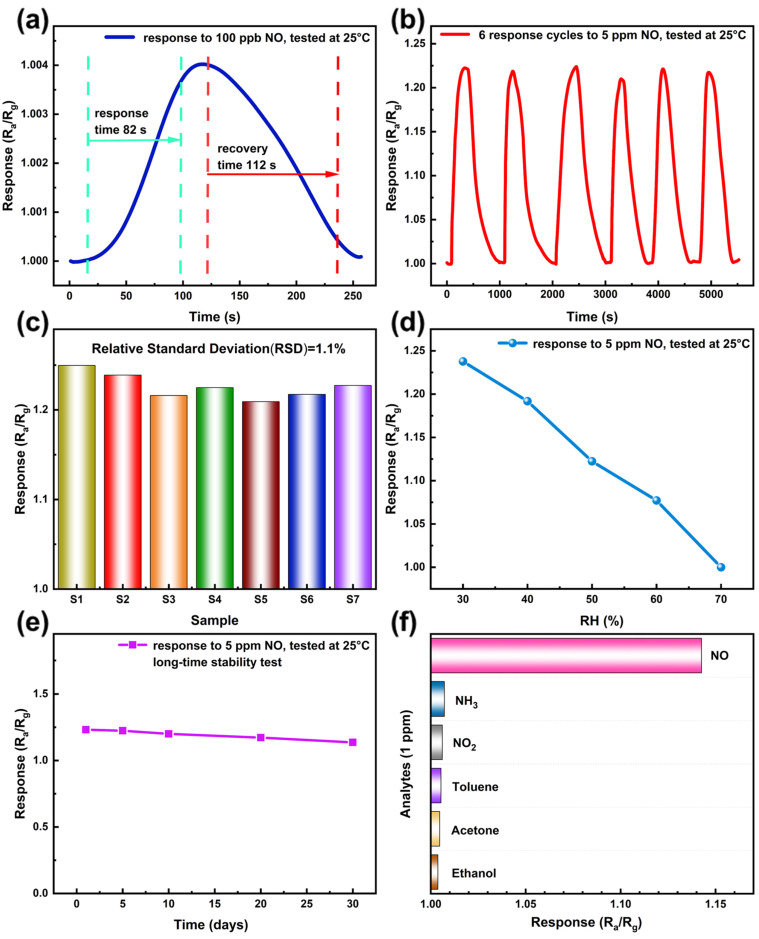
(**a**) A response–recovery curve of the sensor towards 100 ppb NO at room temperature. (**b**–**f**) show the repeatability test, batch-to-batch reproducibility test, relative humidity-dependent test, long-term stability test, and selectivity test of the sensor, respectively.

Figure 7a shows a single response–recovery cycle of the sensor exposed to 100 ppb NO at room temperature. Upon NO exposure, the resistance decreases rapidly, reaching 90% of its steady-state value within 82 s. After the gas supply is terminated, the resistance recovers to 90% of its initial baseline within 112 s. Figure 7b presents the repeatability test of the sensor at 5 ppm NO. The sensor exhibits consistent response values around 1.22, indicating the robust repeatability at higher concentrations. To evaluate practical applicability, the batch-to-batch reproducibility of the sensor was further investigated. Figure 7c displays the response values of seven sensors fabricated from seven different batches of materials synthesized under identical conditions. The sensors exhibit highly comparable response values with a relative standard deviation (RSD) of 1.1%, demonstrating good batch uniformity. Figure 7d shows the sensor’s responses to 5 ppm NO at different levels of relative humidity (RH). As RH increases, the response decreases significantly, which can be attributed to the adsorption of water molecules at high humidity. Water molecules can adsorb onto the nanofiber surface and displace pre-adsorbed oxygen species. This competitive adsorption between water and oxygen reduces the number of available active sites on the nanofibers, thereby decreasing the response to NO [42]. Long-term stability tests conducted over a 30-day period are presented in Figure 7e. The response value decreased by 5.9% on the 30th day compared with that on the first day, demonstrating stable and consistent sensor performance. Possible strategies to mitigate this humidity-induced sensitivity loss include surface modification with hydrophobic coatings to reduce water adsorption, incorporation of humidity compensation algorithms or circuits, and periodic thermal or UV treatment to restore the active sites. These approaches could improve the long-term stability and humidity tolerance of the sensor and will be considered in future work. Selectivity, another key performance metric, was assessed using reference gases including NH_3_, NO_2_, toluene, acetone, and ethanol, each at a concentration of 1 ppm and tested at 25 °C. As shown in Figure 7f, the response to NO is the highest, compared with the other test gases, highlighting the good selectivity of the sensor toward NO. All response curves are provided in the Supplementary Materials. Table 2 presents a performance comparison of NO gas sensors based on various materials. It should be noted that the response definitions (Ra/Rg or Rg/Ra), operating temperatures, and tested concentration ranges reported in the literature are not fully consistent; these differences are explicitly indicated in Table 2 and should be taken into account when interpreting the comparative performance. In contrast to many previously reported sensors that require elevated operating temperatures and/or relatively higher target gas concentrations for effective NO detection, the present Pt/WO_3_-CNF-based sensor achieves reliable NO detection at room temperature (25 °C) with an experimental detection limit of 100 ppb. This combination of room-temperature operation, high-ppb-level NO sensing capability, and simple device configuration places the proposed sensor among NO sensors with comparatively lower reported detection limits under room-temperature operating conditions.

### 3.3. Sensing Mechanism Analysis

**Figure 8:**
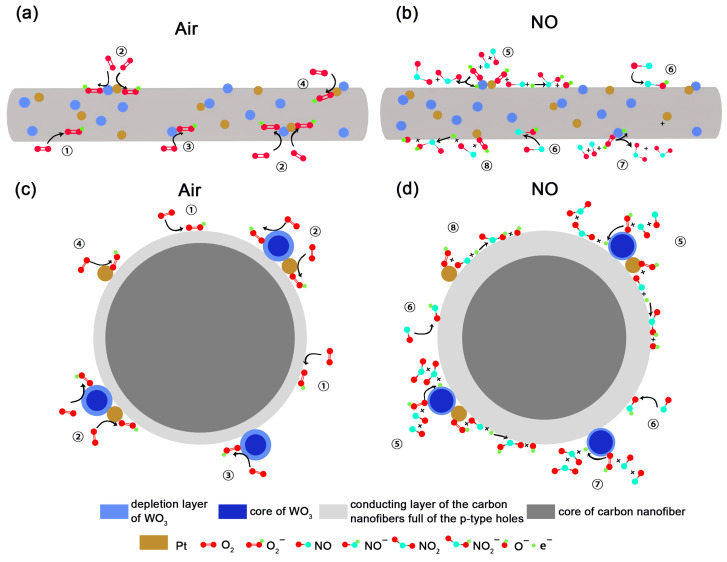
Schematic illustration of the gas-sensing mechanism of the Pt/WO_3_-CNF composite. (**a**,**c**) show the sensor is in air, and (**b**,**d**) in the atmosphere of NO.

Nitric oxide (NO) is generally considered a weak reducing gas in metal oxide–based gas sensors, which in our Pt/WO_3_-CNF system results in a decrease of the sensor resistance. As described in Equation (2) and Figure 8a,c, under ambient air, O_2_ molecules adsorb on the sensor surface and extract electrons from CNF (➀ in Figure 8c), WO_3_ (➂ and ➁ in Figure 8c), and Pt (➃ and ➁ in Figure 8c), yielding ionosorbed $O_{2}^{-}$ species. Because WO_3_ is an *n*-type metal-oxide semiconductor, electron withdrawal generates a surface depletion layer and thus increases its surface resistance. In contrast, CNF is *p*-type, with holes as the majority carriers; electron extraction from CNF increases hole concentration and thereby decreases its surface resistance. In addition, Pt exhibits strong catalytic activity toward oxygen and promotes the ionosorption of $O_{2}^{-}$ on CNF via the well-known spillover effect [77]. In this mechanism, Pt and Pt/WO_3_ decorating clusters provide sites for dissociative O_2_ adsorption and pathways for the migration of oxygen adatoms onto the CNF surface, increasing the population of highly reactive atomic oxygen species on the modified surface [78].

As shown in Equation (3) and Figure 8b,d, exposure to NO leads to adsorption on CNF and further electron withdrawal, producing ${\mathrm{NO}}_{\left(\mathrm{ads}\right)}^{-}$ (➇ in Figure 8d). This additional electron extraction raises the hole concentration and further lowers the resistance. Moreover, ${\mathrm{NO}}_{\left(\mathrm{gas}\right)}$ reacts with $O_{2\left(\mathrm{ads}\right)}^{-}$ to form ${\mathrm{NO}}_{2\left(\mathrm{ads}\right)}^{-}$ and $O_{\left(\mathrm{gas}\right)}^{-}$ (Equation (4); ➇ and ➄ in Figure 8d) [79]. In this reaction, NO again accepts electrons from the CNF surface, generating additional holes and reinforcing the resistance drop; the concomitant consumption of $O_{2,\left(\mathrm{ads}\right)}^{-}$ amplifies electron extraction as in Equation (2). Finally, as indicated by Equation (5) and by ➆ and ➄ in Figure 8d, on the WO_3_ surface NO reacts with $O_{2\left(\mathrm{ads}\right)}^{-}$ to produce NO_2_ while releasing electrons back to WO_3_, which decreases the surface resistance of WO_3_ [80].

(2) $$\begin{array}{cc} O_{2(\mathrm{ads})}+e^{-} & \;\to \;{O_{2}^{-}}_{\left(\mathrm{ads}\right)} \end{array}$$

(3) $$\begin{array}{cc} {\mathrm{NO}}_{\left(\mathrm{gas}\right)}+e^{-} & \;\to \;{{\mathrm{NO}}^{-}}_{\left(\mathrm{ads}\right)} \end{array}$$

(4) $$\begin{array}{cc} {\mathrm{NO}}_{\left(\mathrm{gas}\right)}+{O_{2}^{-}}_{\left(\mathrm{ads}\right)}+e^{-}\; & \to \;{{\mathrm{NO}}_{2}^{-}}_{\left(\mathrm{ads}\right)}+{O^{-}}_{\left(\mathrm{ads}\right)} \end{array}$$

(5) $$\begin{array}{cc} 2{\mathrm{NO}}_{\left(gas\right)}+{O_{2}^{-}}_{\left(\mathrm{ads}\right)}\; & \to \;2{\mathrm{NO}}_{2(gas)}+e^{-} \end{array}$$

The enhanced gas sensing performance of the sensor can be attributed to the following two factors.

**Intrinsic properties of CNF:**

Abundant adsorption sites. The sp^2^-hybridized carbon framework of CNF provides numerous active sites for NO adsorption, significantly improving sensitivity [81,82]. Oxygen-containing functional groups. CNF retains residual oxygen functionalities [83], such as carboxyl (-COOH) and hydroxyl (-OH) groups. These groups interact with NO molecules through adsorption or chemical reactions, thereby enhancing both selectivity and sensitivity. π-conjugated structure. The π-conjugated system of carbon materials enables strong interactions with NO, facilitating detection at very low concentrations [84,85].

**Heterojunction amplification and synergistic pathways:**

The p–n heterojunction behaves as a gate, where minor variations in surface charge induced by NO adsorption lead to significant modulation of the depletion region, resulting in pronounced changes in resistance. Furthermore, CNFs serve as a highly conductive backbone enabling rapid hole transport, while WO_3_ and Pt nanoparticles contribute a high surface area and abundant active oxygen species. These components act synergistically, yielding higher sensitivity.

### 3.4. NO Sensing Performance in Simulated Breath Exhaled Samples

Simulated breath samples were prepared by introducing exogenous NO at concentrations of 100, 150, and 200 ppb into Tedlar bags containing exhaled breath collected from healthy volunteers. Considering the pronounced influence of humidity on the sensor response, all gas samples were dried prior to measurement. To better reflect practical operating conditions, breath samples from two different healthy individuals were selected, and exogenous NO was introduced into each breath matrix to construct simulated breath environments for sensor performance evaluation.

**Figure 9:**
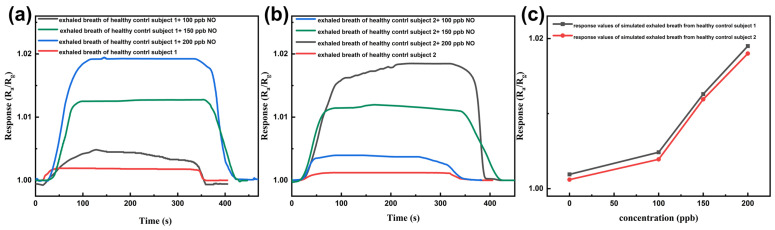
(**a**,**b**) Dynamic response curves of the sensor toward simulated breath samples derived from two healthy volunteers; (**c**) Relationship between the sensor response and the concentration of exogenous NO introduced into the breath samples.

Figure 9a,b present the dynamic response curves of the sensor toward these simulated breath samples. Distinguishable response variations were observed as the NO concentration increased, indicating that the sensor is capable of responding to changes in NO concentration within a complex breath background. Figure 9c summarizes the response values obtained from Figure 9a,b. The horizontal axis represents the concentration of exogenous NO added to the Tedlar bags, while the zero point corresponds to the original breath samples from healthy volunteers without added NO.

A slight variation in baseline response was observed among breath samples from different healthy individuals, which could be related to differences in endogenous NO content or the presence of other accompanying gaseous species. With increasing NO concentration, the sensor response increased accordingly. These observations indicate that the proposed sensor is capable of resolving different NO levels in simulated breath samples and may serve as a useful experimental platform for further studies under more representative breath conditions.

## 4. Conclusions

In this study, Pt/WO_3_ co-decorated carbon nanofibers (CNFs) were synthesized via electrospinning. The morphology, microstructure, and elemental composition were characterized using SEM, HRTEM, XRD, XPS, and EDS analyses. The sensor based on CNFs co-decorated with 1.07 at% WO_3_ and 0.07 at% Pt exhibited the highest gas-sensing performance toward NO detection, achieving an experimentally demonstrated detection limit of 100 ppb at room temperature (25 °C). It demonstrated a rapid response, excellent repeatability, good batch-to-batch reproducibility, and stable performance over 30 days. The enhanced sensing behavior can be attributed to the abundant adsorption sites, oxygen-containing functional groups, π-conjugated structure, and a highly conductive backbone of the CNFs, as well as the high surface area, rich active oxygen species, and synergistic effects introduced by the WO_3_ and Pt nanoparticles. Under controlled laboratory conditions and simulated breath environments derived from healthy volunteers, the proposed sensor demonstrates potential as a room-temperature NO sensing platform. Although validation using real clinical breath samples is beyond the scope of this study, the present results provide a materials-level basis for further optimization and evaluation of NO sensing technologies relevant to breath NO analysis.

## Figures and Tables

**Table 1:** The gas sensing performance of the sensor, which was tested at 25 °C towards a series of concentrations of NO.

NO Concentration	100 ppb	200 ppb	500 ppb	1 ppm	5 ppm	10 ppm	20 ppm	25 ppm
Response (Ra/Rg)	1.004	1.021	1.038	1.144	1.214	1.239	1.398	1.440
Response Time (s)	82	104	108	111	134	58	73	97
Recovery Time (s)	112	277	412	427	322	370	284	238

**Table 2:** A performance comparison of NO gas sensors based on various materials. Ope. T and Con. denote operating temperature and concentrations. R.T. denotes room temperature.

Materials	Ope. T (°C)	Con. (ppm)	Sensor Response	$T_{\mathrm{res}}/T_{\mathrm{rec}}$ (s)	Detection Limit (ppm)	Reference
PtO_2_/SnO_2_ nanoparticles	150	5	2640 (Rg/Ra)	126/1500	0.125	[53]
ZnO tube bundles	92	10	37 (Rg/Ra)	40/12	0.1	[63]
WO_3_ nanorods	150	10	2.29 (Rg/Ra)	56/79	–	[64]
Coralline-like ZnO	R.T.	40	23.59 (Rg/Ra)	331/1285	5	[65]
Graphene/ZnO nanowires	70	50	0.853 (Ra/Rg)	725/414	50	[66]
Pd@Fe_2_O_3_/MWCNTs/WO_3_	25	0.5	1.18 (Rg/Ra)	291/511	0.1	[67]
Tb_2_O_3_/ZnO Nanofilms	180	1	28.3 (Rg/Ra)	208/248	0.01	[59]
Pd-WO_3_	200	20	82 (Rg/Ra)	27/23	5	[68]
In_2_O_3_	200	20	10.3 (Rg/Ra)	10/360	2	[69]
NiO/SnO_2_	R.T.	2.5	0.982 (Rg/Ra)	–	2.5	[70]
Co-TCPP(Fe)/Ti_3_C_2_Tx	R.T.	10	2 (Ra/Rg)	95/15	0.2	[71]
SnO_2_ nanotubes	160	0.5	33.3 (Rg/Ra)	214/115	0.01	[72]
N-rGO	R.T.	1	1.7 (Rg/Ra)	–	0.4	[73]
TiO_2_-rGO	30	2.75	1.07 (Rg/Ra)	440/–	–	[74]
N-rGO/ZnO	90	0.8	23 (Rg/Ra)	284/473	0.1	[75]
ZnO/CdO nanofibers	215	33	22.6 (Rg/Ra)	35/630	1.2	[76]
Pt/WO_3_-CNF	R.T.	5	1.24 (Ra/Rg)	176/439	0.1	This work

## Data Availability

The original contributions presented in this study are included in the article/Supplementary Materials. Further inquiries can be directed to the corresponding authors.

## Supplementary Materials

The following supporting information can be downloaded at: https://www.mdpi.com/article/10.3390/s26031069/s1, Figure S1: The XPS full spectrum of the Pt/WO_3_-CNF sensing material; Figure S2: (a–e) The real-time response variation of the sensor with the different ratio of WO_3_, corresponding to Figure 6a in the text; Figure S3: (a–e) The real-time response variation of the sensor with the different ratio of Pt, corresponding to Figure 6b in the text; Figure S4: (a–e) The real-time response variation of the sensor with the different temperature, corresponding to Figure 6c in the text; Figure S5: (a–g) The real-time response variation of the sensor towards different concentrations of NO (200-2500 ppb) at room temperature, corresponding to Figure 6e in the text; Figure S6: (a–g) The real-time response variation of the sensor with different batches of sample, corresponding to Figure 7c in the text; Figure S7: (a–e) The influence of humidity on the response of the sensor, corresponding to Figure 7d in the text; Figure S8: (a–e) The long-term stability test of the sensor, corresponding to Figure 7e in the text; Figure S9: (a–e) The selectivity test of the sensor towards reference gas, corresponding to Figure 7f in the text; Figure S10: Selected-area electron diffraction (SAED) pattern of the Pt/WO_3_–CNF composite. The pattern is dominated by diffuse scattering from the amorphous CNF matrix. Faint and discontinuous ring-like features can be observed, suggesting the presence of ultrasmall crystalline domains with very low content embedded in the CNF support.
