# Supplementary material for: A Room-Temperature, High-ppb-Level NO Gas Sensor Based on Pt/WO3 Co-Decorated Carbon Nanofibers Towards Asthma-Relevant Breath Analysis Application
Source: Sensors (Basel). 2026 Feb 6;26(3):1069. doi: 10.3390/s26031069 (PMC12900128; doi:10.3390/s26031069)
Supplement: Supplementary file 1 [file sensors-26-01069-s001.zip › sensors-4127545-supplementary.pdf]

Supporting Information

A Room-Temperature, ppb-Level NO Gas Sensor Based on Pt/WO_3 Co-Decorated Carbon Nanofibers towards Asthma-Relevant Breath Analysis Application

Shanshan Yu[1], Xingyu Liu[1], Jinshun Wang[1], Qiuxia Li[1], Yuhao Pang[1], Lixin Zhang[1], Chen Yang[1], Qingkuan Meng[1], Cao Wang[1], Qiang Jing[1]*, Jingwei Chen[1]*, and Bo Liu[2]

[1]Laboratory of Functional Molecules and Materials, School of Physics and Optoelectronic Engineering, Shandong University of Technology, 266 Xincun Xi Road, Zibo 255000, China.

[2]School of Mathematics and Physics, Xi' an Jiaotong-Liverpool University, Suzhou, 215123, China.

*Corresponding author(s). E-mail(s):

jingqiang@sdut.edu.cn;chenjingwei@sdut.edu.cn

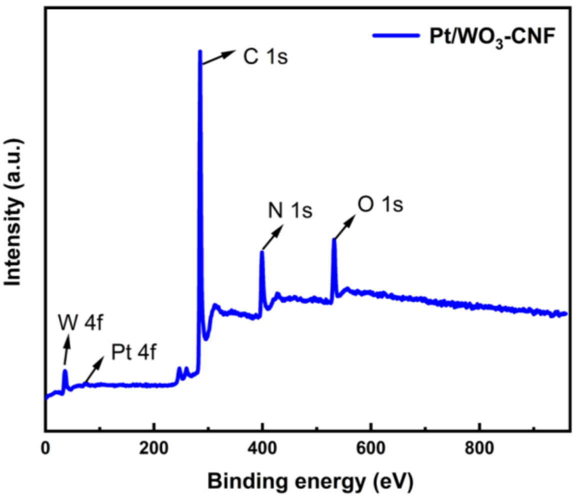

Fig. S1: The XPS full spectrum of the Pt/WO_3-CNF sensing material.

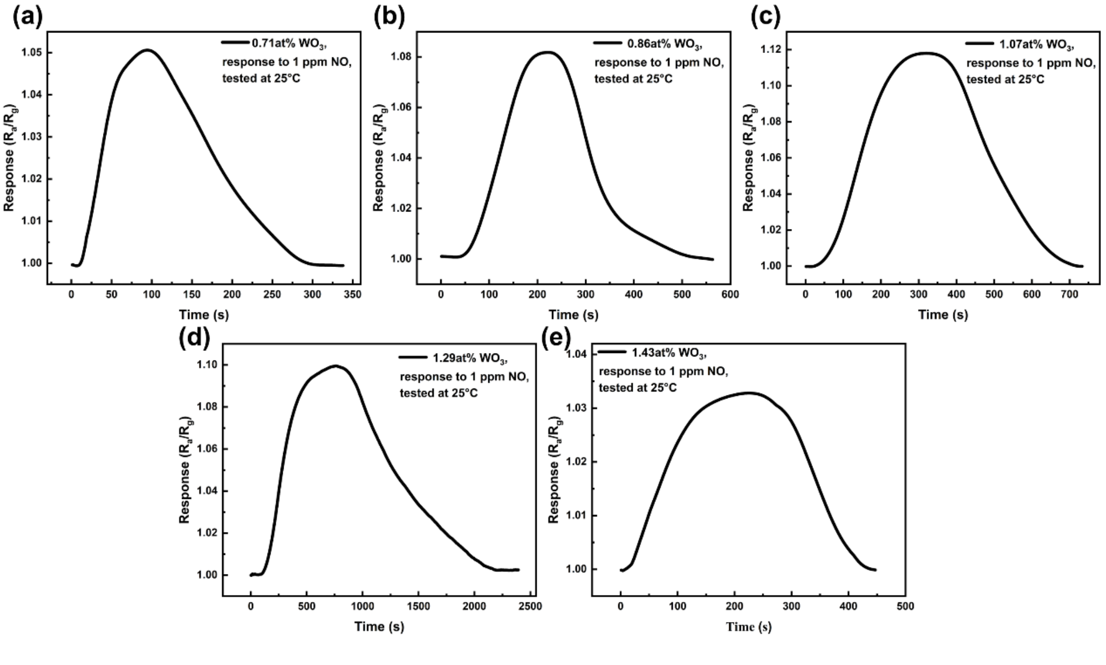

Fig. S2: (a)-(e) The real-time response variation of the sensor with the different ratio of WO_3, corresponding to Fig.6(a) in the text.

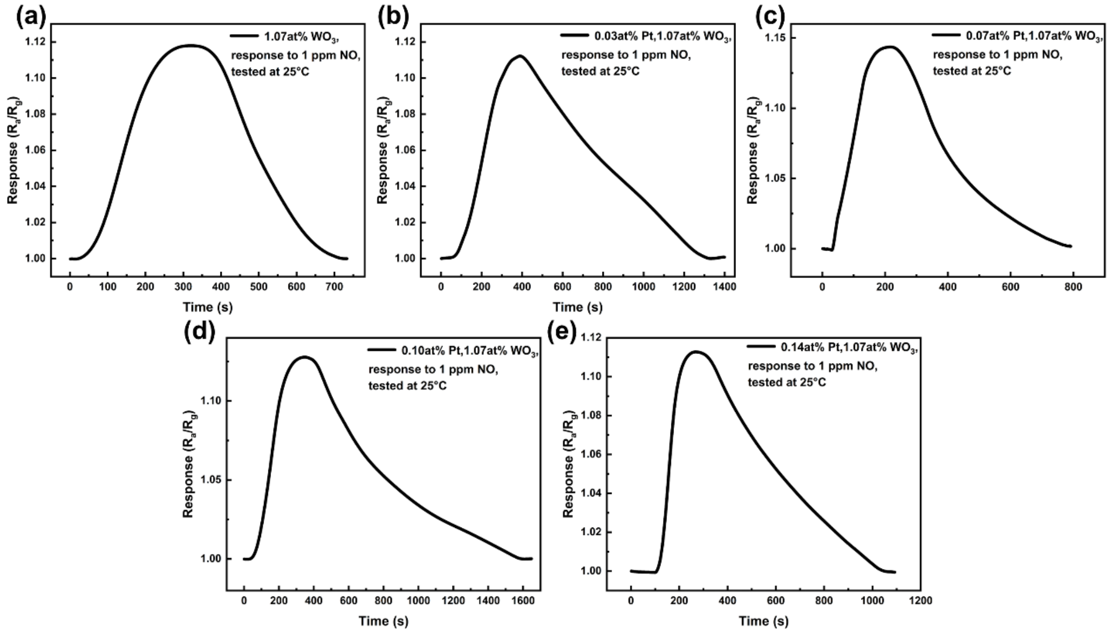

Fig. S3: (a)-(e) The real-time response variation of the sensor with the different ratio of Pt, corresponding to Fig.6(b) in the text.

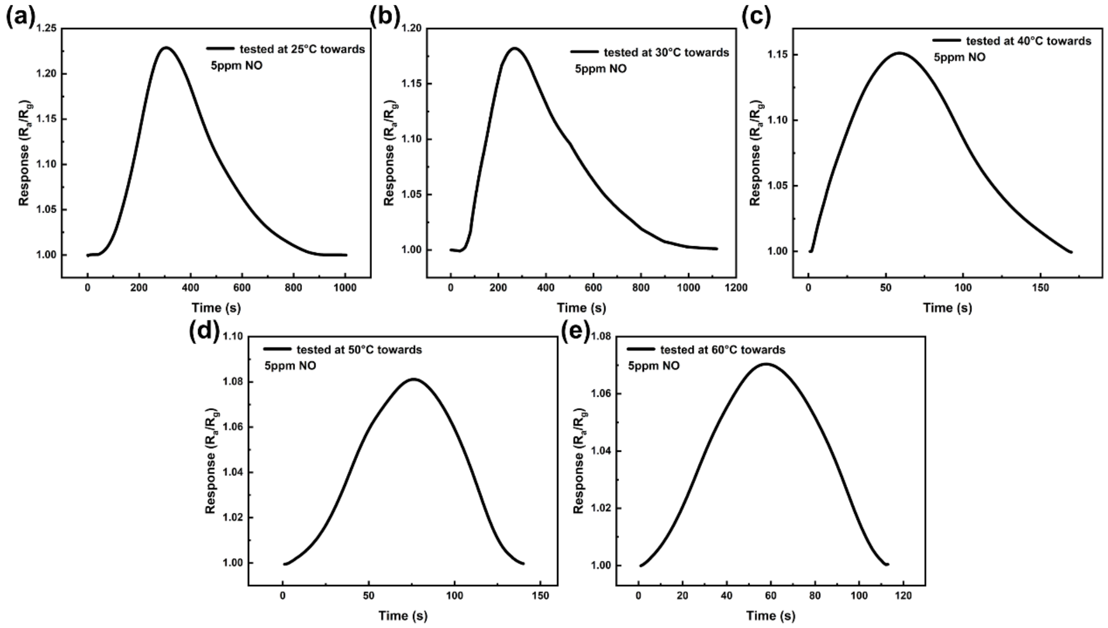

Fig. S4: (a)-(e) The real-time response variation of the sensor with the different temperature, corresponding to Fig.6(c) in the text.

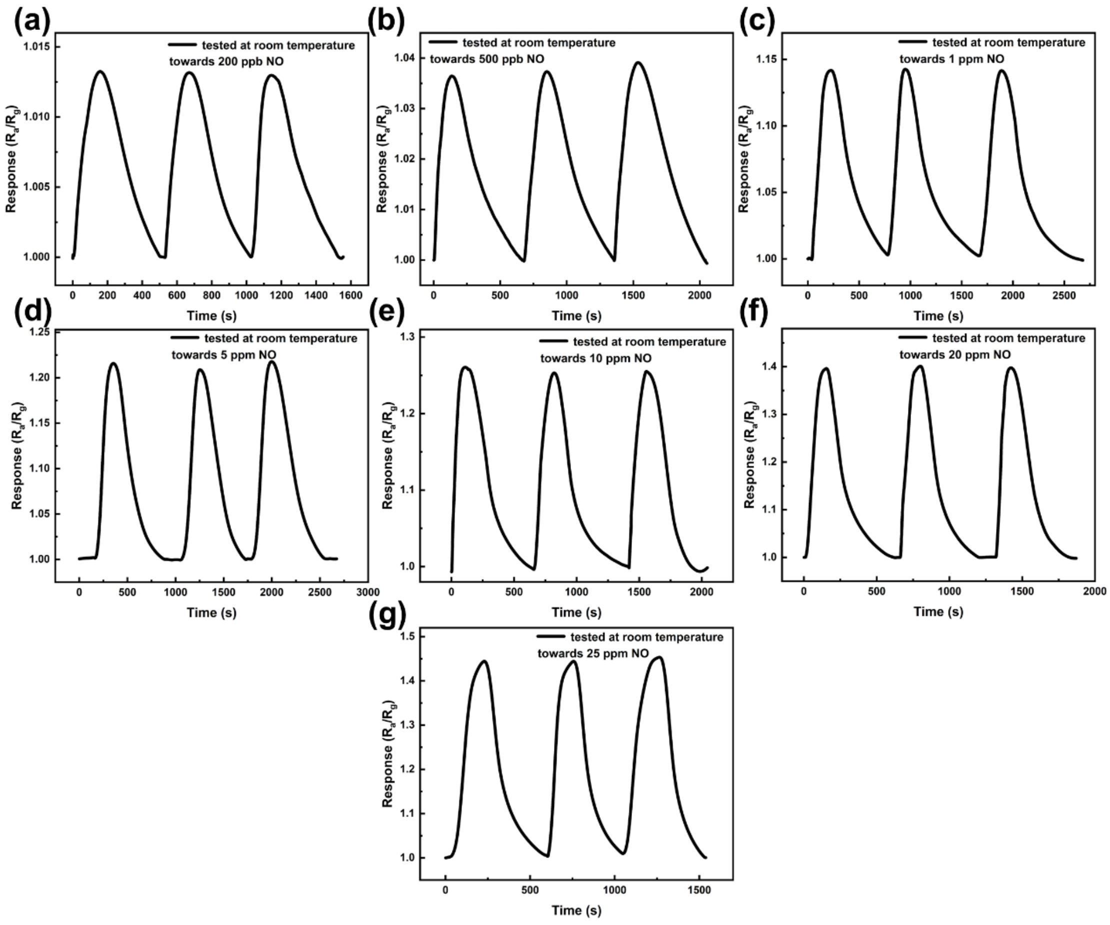

Fig. S5: (a)-(g) The real-time response variation of the sensor towards different concentrations of NO (200-2500 ppb) at room temperature, corresponding to Fig.6(e) in the text.

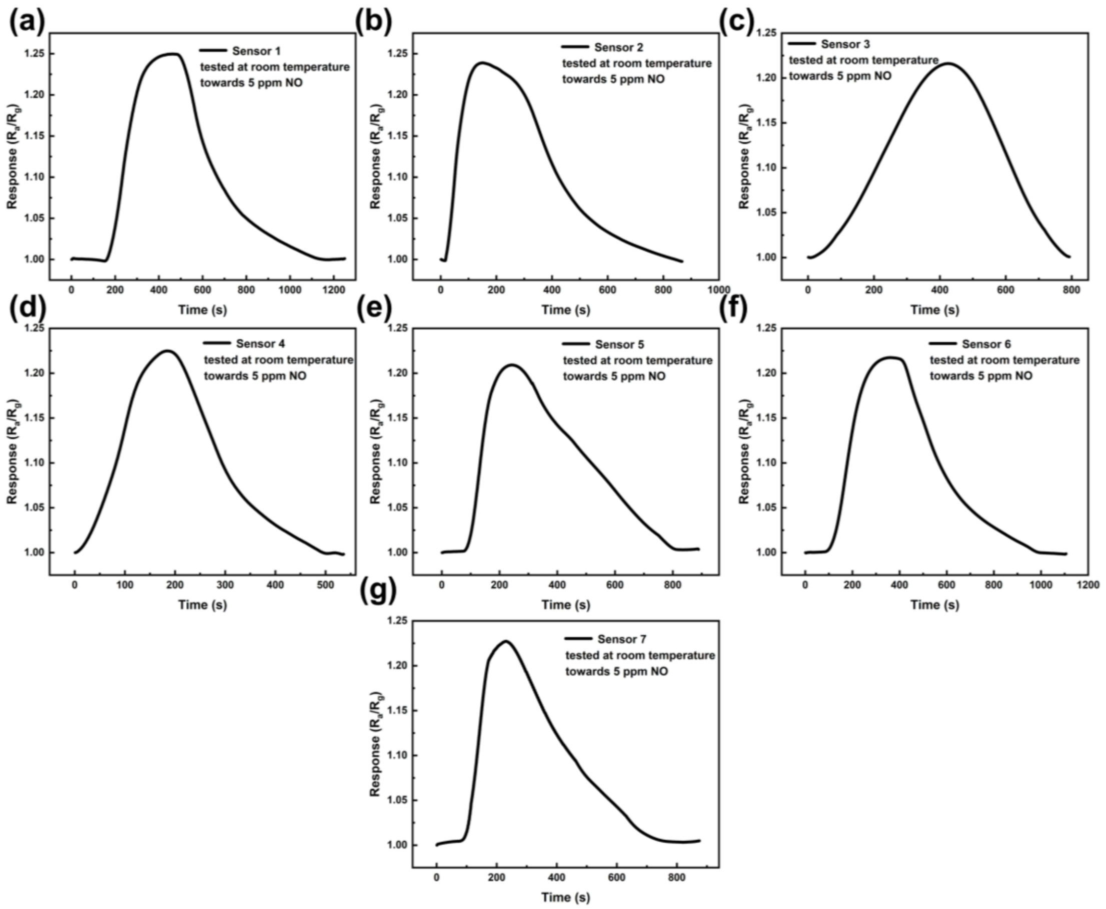

Fig. S6: (a)-(g) The real-time response variation of the sensor with different batches of sample, corresponding to Fig.7(c) in the text.

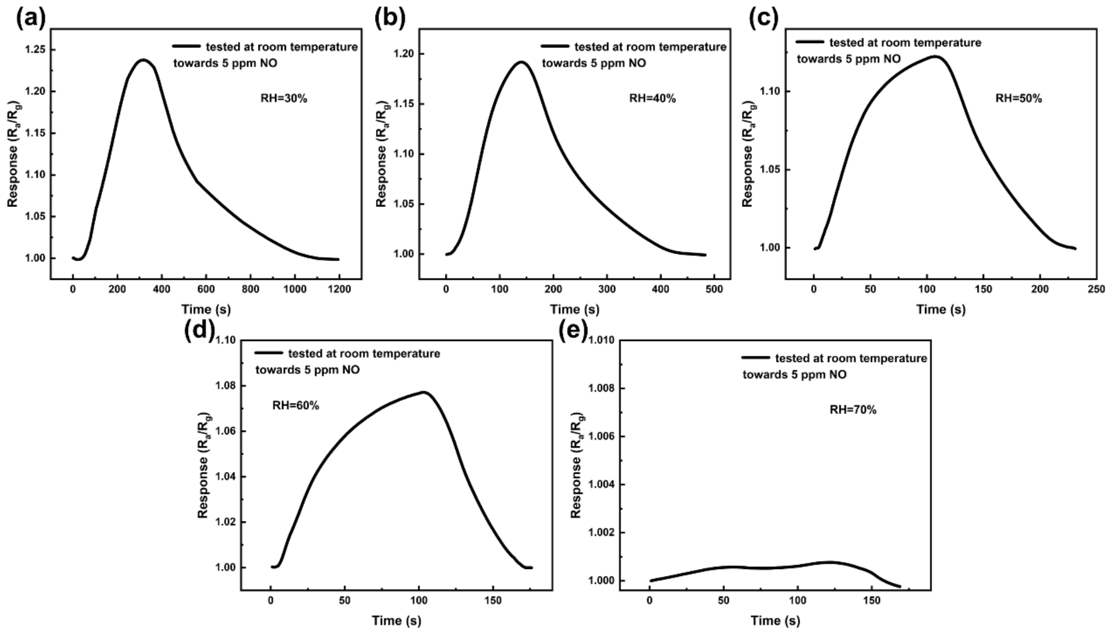

Fig. S7: (a)-(e) The influence of humidity on the response of the sensor, corresponding to Fig.7(d) in the text.

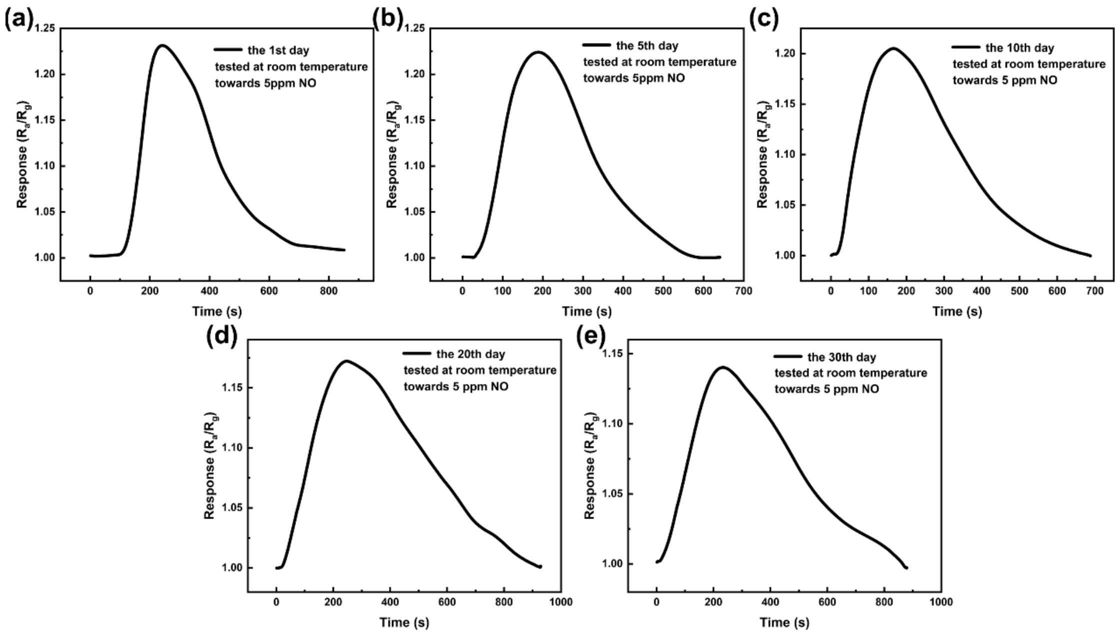

Fig. S8: (a)-(e) The long-term stability test of the sensor, corresponding to Fig.7(e) in the text.

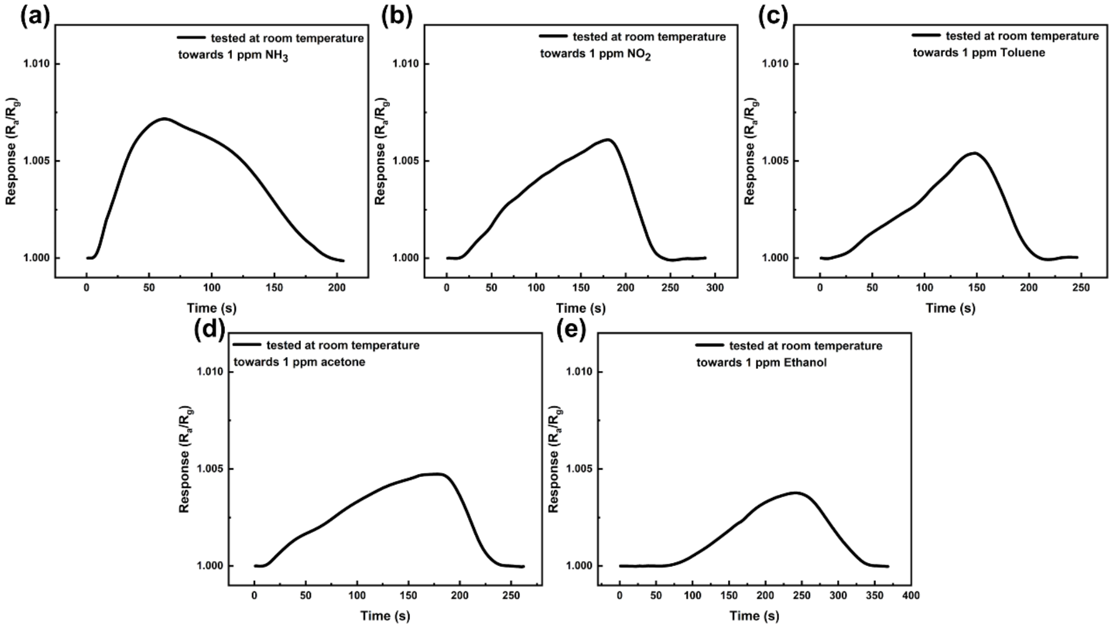

Fig. S9: (a)-(e) The selectivity test of the sensor towards reference gas, corresponding to Fig.7(f) in the text.

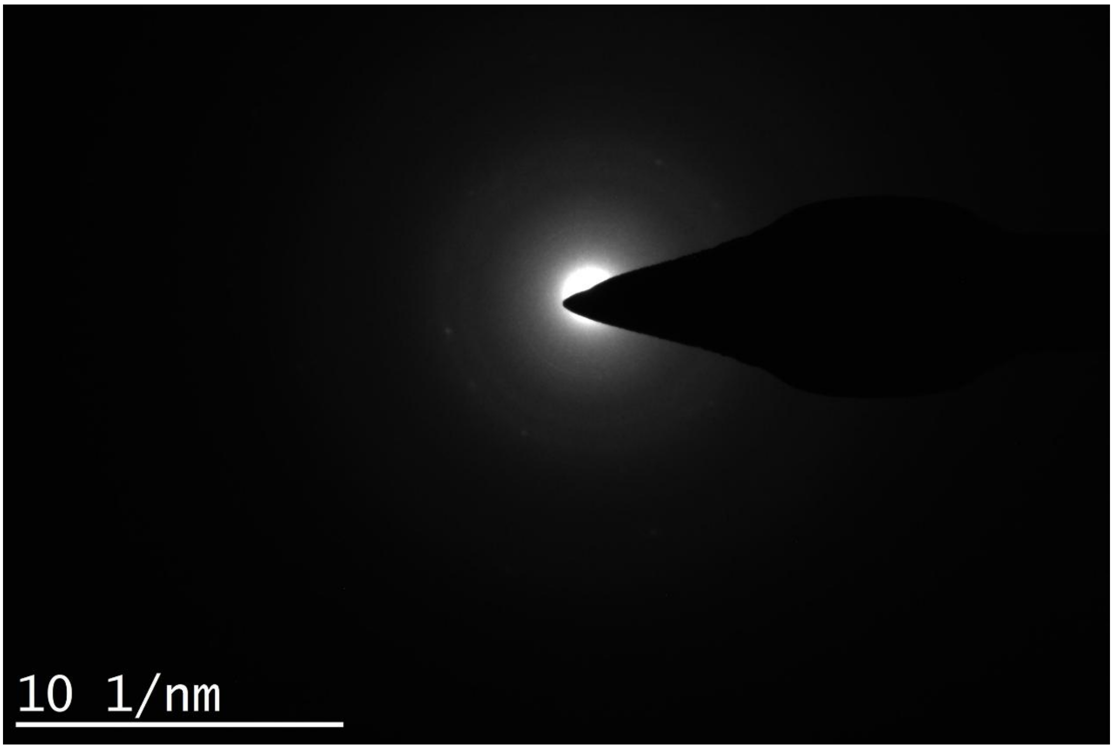

Fig. S10: Selected-area electron diffraction (SAED) pattern of the Pt/WO_3–CNF composite. The pattern is dominated by diffuse scattering from the amorphous CNF matrix. Faint and discontinuous ring-like features can be observed, suggesting the presence of ultrasmall crystalline domains with very low content embedded in the CNF support.
